# Dentistry by the Hour

**Published:** 1874-04

**Authors:** 


*Dentistry by the Hour.*—The *Boston Cammercial Bulletin* comments as follows upon the practice of regulating dental fees by time consumed:

"Our fashionable and 'high-toned' dentists have made a new departure in the conduct of their business. Instead of taking a man's mouth by contract, and charging a lump price for the torture they have inflicted upon him, it is now proposed to do "hour work" exclusively, the price being fixed at five dollars for every sixty minutes' work with forceps and files, to say nothing of those soothing scrapers, with which these gentry delight to fondle a tender tooth. There will be no hurry now, and the patient may rest assured that although 'time is money,' it is, in this instance, his money, to gain which the artist will linger lovingly over each gaping cavity, and by the aid of a delicate steel hook, dally with a tender nerve to while the time away. There is some relief when the washerwoman's boy drops in to bring a fresh supply of napkins, wherewith to smother the profanity that is but too apt to come to the surface in a dentist's chair, especially if the dentist, never dealing in small sums, has nothing smaller than an X with him, and is compelled to visit a neighbor for the change; but even the comfort which this respite brings loses its charms when the victim reflects that these minutes—precious indeed—are just as costly as though the dentist were subjecting him to the most ingenious and exquisite torture."

